# Compared to Palliative Care, Working in Intensive Care More than Doubles the Chances of Burnout: Results from a Nationwide Comparative Study

**DOI:** 10.1371/journal.pone.0162340

**Published:** 2016-09-09

**Authors:** Sandra Martins Pereira, Carla Margarida Teixeira, Ana Sofia Carvalho, Pablo Hernández-Marrero, InPalIn

**Affiliations:** 1 Instituto de Bioética, Universidade Católica Portuguesa, Porto, Portugal; 2 Hospital de Santo António, Centro Hospitalar do Porto, Porto, Portugal; 3 Instituto de Ciências Biomédicas Dr. Abel Salazar, Universidade do Porto, Porto, Portugal; Azienda Ospedaliero Universitaria Careggi, ITALY

## Abstract

**Introduction:**

Professionals working in intensive and palliative care units, hence caring for patients at the end-of-life, are at risk of developing burnout. Workplace conditions are determinant factors to develop this syndrome among professionals providing end-of-life care.

**Objectives:**

To identify and compare burnout levels between professionals working in intensive and palliative care units; and to assess which workplace experiences are associated with burnout.

**Methods:**

A nationwide, multicentre quantitative comparative survey study was conducted in Portugal using the following instruments: Maslach Burnout Inventory–Human Services Survey, Questionnaire of workplace experiences and ethical decisions, and Questionnaire of socio-demographic and professional characteristics. A total of 355 professionals from 10 intensive care and 9 palliative care units participated in the survey. A series of univariate and multivariate logistic regression analyses were performed; odds ratio sidelong with 95% confidence intervals were calculated.

**Results:**

27% of the professionals exhibited burnout. This was more frequent in intensive care units (OR = 2.525, 95% CI: 1.025–6.221, p = .006). Univariate regression analyses showed that higher burnout levels were significantly associated with conflicts, decisions to withhold/withdraw treatment, and implementing palliative sedation. When controlling for socio-demographic and educational characteristics, and setting (intensive care units versus palliative care units), higher burnout levels were significantly and positively associated with experiencing conflicts in the workplace. Having post-graduate education in intensive/palliative care was significantly but inversely associated to higher burnout levels.

**Conclusions:**

Compared to palliative care, working in intensive care units more than doubled the likelihood of exhibiting burnout. Experiencing conflicts (e.g., with patients and/or families, intra and/or inter-teams) was the most significant determinant of burnout and having post-graduate education in intensive/palliative care protected professionals from developing this syndrome. This highlights the need for promoting empowering workplace conditions, such as team empowerment and conflict management. Moreover, findings suggest the need for implementing quality improvement strategies and organizational redesign strategies aimed at integrating the philosophy, principles and practices of palliative care in intensive care units.

## Introduction

Professionals working in intensive and palliative care units, hence caring for patients suffering from a life-threatening disease, sometimes at the end-of-life, are at risk of developing work-related problems, such as burnout. Burnout is a syndrome of exhaustion related to the job. It is characterized by physical and emotional fatigue, and is usually related to work stress and dedication to a cause, a way of life that does not match the person’s expectations [1]. Burnout syndrome is widely described in its tri-dimensionality: (i) Emotional exhaustion (EE); (ii) Depersonalization (DEP); and (iii) (reduced sense of) Personal and Professional Accomplishment (PPA) [2,3].

High incidences of burnout were described in helping professions due to the establishment of intense interpersonal relationships [4]. In the context of healthcare, particularly in intensive and palliative care settings, professionals are often confronted with very complex and highly demanding situations (e.g., end-of-life decisions, communication about difficult issues such as life-threatening diagnosis and limited prognosis, family conflicts, human suffering and vulnerability), which, together with disempowering conditions of the workplace (e.g., work overload, role ambiguity, conflicts), can be a source of considerable stress, emotional constraint, moral distress, disempowerment and burnout [1,4–7]. According to several authors [2,3,8,9], these risk factors can be organized into four main dimensions: personal characteristics, organizational factors, quality of working relationships and exposure to end-of-life issues. Organizational and services’ negative working culture, in which perceived burnout complaints among professionals are common, might create a “contagion effect” among professionals [10].

Evidence shows that working in intensive care units (ICUs) is associated to higher burnout levels and it may vary across countries [4,8–15]. In fact, a recent systematic review showed that the prevalence of burnout among healthcare professionals in ICUs varied from 0% to 70.1% with this syndrome being associated to a broad range of variables, such as the work environment, professional role and conflicts [16]. In the United States of America, a recent report issued by the Critical Care Societies Collaborative highlighted that up to 45% of the critical care physicians and nearly a third (25% to 33%) of critical care nurses in this country were experiencing severe burnout [9].

A Portuguese study conducted with nurses and physicians working in ICUs found that 31% out of 300 respondents exhibited burnout [4]. The authors concluded that higher burnout levels were associated to organizational factors, workload and workplace experiences, such as making ethical decisions [4,17]. Similar findings were reported elsewhere, indicating that organizational factors are indeed associated with higher burnout levels in ICUs [9,11,12,14,16]. Experiencing conflicts (e.g., with patients and/or families, intra and/or inter-teams) is also a determinant variable of burnout [8,14]. Furthermore, it seems that professional groups are affected in diverse manners: while nurses are more affected in their sense of exhaustion and accomplishment, physicians are affected mostly in terms of depersonalization [4].

Contrarily, working in palliative care units (PCUs) is not associated to higher burnout levels [1], especially when compared to other work contexts. A study conducted with nurses and physicians working in PCUs in Portugal indicated that only 3% of professionals exhibited this syndrome. The majority of the respondents were at low (55%) and medium risk (30%) of burnout [5,18]. Diverse findings, however, were reported in other countries. A significant prevalence of burnout was identified, for instance, among hospice and palliative care (PC) practitioners in Singapore, being associated with working more hours and in different work settings [19]. Effective coping mechanisms like physical well-being, setting boundaries, transcendental meditation and quiet reflection, passion for one’s work, realistic expectations, remembering patients, rituals after a patient’s death, inter-professional team-based communication and strategies, and organizational activities were associated with less burnout in palliative care [1,5,18,19]. The mediating role of coping styles in the development of burnout has been highlighted in other healthcare settings, suggesting the need to implement active strategies aiming at enhancing professional performance [20].

Several differences and similarities can be found in the workplace experiences faced by professionals working in ICUs and PCUs. On the one hand, while in intensive care (IC) the major goal is to save lives and professionals feel they have the patient’s life “in their hands”, in palliative care (PC) the major purpose is to promote the quality of life, minimize suffering and provide a peaceful death. This may have an effect in the way professionals cope with the demanding situations of their daily practice. On the other hand, professionals working in ICUs and in PCUs care for patients with life-threatening diseases or conditions, provide end-of-life care, make ethical decisions, and face human vulnerability, suffering, dying and death. Beyond these experiences, in both ICUs and PCUs as in many other healthcare settings, professionals face other common experiences, such as communication issues, different perspectives about the patients’ best interest and conflicts that may affect their emotional well-being and influence the quality of care. Furthermore, particularly nowadays, due to the economic crisis, financial constrains cause resource shortages and work overload in healthcare services, which can contribute to physical and emotional exhaustion [1,3,4,7–20].

Despite the broad spectrum of literature about burnout in healthcare professions, there is a lack of specific studies comparing burnout among professionals working in intensive and palliative care units. To our knowledge, this comparison has never been investigated thoroughly, even less in Portugal. Moreover, when looking at the existing evidence on burnout in intensive and palliative care, findings lack of consistency; therefore, justifying the need to study this phenomenon.

The objectives of our study are: (i) To identify and compare burnout levels between professionals working in ICUs and PCUs; and (ii) to assess which workplace experiences are significantly associated with burnout among these professionals.

## Methods

A nationwide, multicentre cross-sectional quantitative survey study was conducted in Portugal.

### Survey instruments

1. A questionnaire of socio-demographic variables and data on professional background (e.g., post-graduate education in intensive care/palliative care).
2. A questionnaire, based on Embriaco et al [12], with a set of workplace experiences in the week prior to and day of questionnaire completion, including workload and conflicts (intra-team, inter-team, with a superior, with patients, with family members).
3. A questionnaire on ethical decisions in the week prior to and day of questionnaire completion, including withholding/withdrawing treatment; palliative sedation; communication and information disclosure of the diagnosis and prognosis to a patient/family.
4. The Maslach Burnout Inventory—Human Services Survey (MBI-HSS). This is a self-report questionnaire that comprises a total of 22 items, corresponding to the three burnout sub-dimensions defined by Maslach et al [21–25]: Emotional Exhaustion (EE) with nine items; Depersonalization (DEP) with five items; and Personal and Professional Accomplishment (PPA) with eight items. The MBI-HSS asks respondents to indicate on a seven-point Likert scale the frequency of experiencing certain feelings related to their work. The MBI-HSS is scored according to the presence and severity of Emotional Exhaustion (EE), Depersonalization (DEP), and reduced sense of Personal and Professional Accomplishment (PPA). This is the most common, widely described and internationally validated instrument used to assess all the three dimensions of burnout. The MBI-HSS has been found to be reliable, valid, and easy to administer [24].

A Portuguese cross-culturally adapted and validated version of the MBI-HSS was used [26], which is aligned with recent international methodological recommendations in this field [27]. Cut-off scores were defined for each dimension and we adopted the following internationally established definition of burnout: high levels of EE and DEP combined with low PPA [3,21–26]. Both the cut-off scores and the definitions of burnout were cross-culturally adapted and validated into the Portuguese context [26]. The risk of burnout was defined as follows: high risk, two of the three dimensions beyond the cut-off point; average risk, one of the three dimensions beyond the cut-off point; low risk, average or low levels in the dimensions EE and DEP, and high or average levels in PPA [24–26,28,29]. For the present statistical analysis, a high level of burnout (high burnout) was defined as the sum of being both in burnout and with high risk of burnout [4,18,22–24,26,28–30].

### Setting, participants, data collection and ethical approval

**Fig 1:**
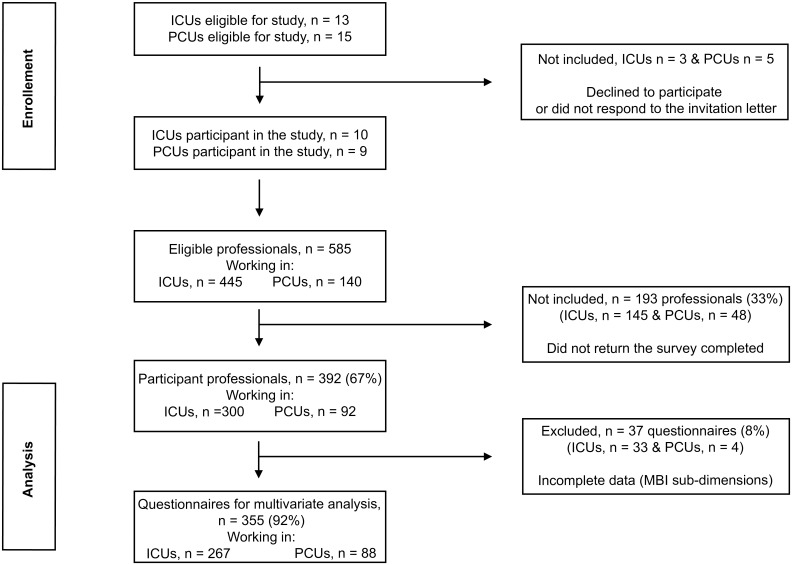
Study flowchart. This figure illustrates all the steps followed from participants’ enrollment throughout their inclusion in the analyses.

All 15 Portuguese PC teams designated in the webpage of the Portuguese Association for Palliative Care (Associação Portuguesa de Cuidados Paliativos, APCP) in September 2008, hence recognized as providing a specialist level of palliative care, and 13 ICUs based in state hospitals in the North of Portugal were invited to participate in this study. Nine PCUs (N = 140 physicians and nurses) and ten ICUs (N = 445 physicians and nurses) accepted this invitation (Fig 1). PCUs were geographically dispersed, covering the North, Centrum and South regions of the country. ICUs were polyvalent and the median Simplified Acute Physiology Score II score at admission was 45. The final sampling frame (i.e., a list of all physicians and nurses in the target population) was 585. These physicians and nurses from all participant PCUs and ICUs were invited to complete the survey.

The study was presented during team-meetings at all participant units where the self-administered questionnaires were distributed. Professionals who were absent or on shift work were handed questionnaires by the head nurse of the unit at a separate time. Completed surveys were inserted into an envelope-box in each unit and collected by the researchers. Participation was anonymous and voluntary. Data collection occurred from October 2008 throughout December 2009.

Ethical approval was obtained from the ethics and scientific committees of the Instituto de Bioética, Universidade Católica Portuguesa. Institutional and ethical approvals were also obtained from all participant institutions, together with the informed consent of each team and participant. Participation was anonymous and voluntary. Data were analyzed and presented with full anonymity.

### Statistical analysis

Data analysis was performed using proper software (SPSS version 21.0). Summary statistics were applied as appropriate. Categorical variables were described through absolute frequencies (n) and relative (%) ones. As the distribution of continuous variables was asymmetric, they were described using the median together with 25^th^ and 75^th^ percentiles. Due to the different sample sizes and the skewness of distributions, only nonparametric exact tests were applied. A Chi-square (χ^2^) independence test was used to examine the association between categorical variables. The Fisher exact test was used when the expected frequency (in any cell of the contingency table analysis) on the association of two categorical variables was less than five. The Mann-Whitney test was used to test hypotheses concerning continuous variables, since their distribution was asymmetric. Univariate and multivariate logistic regression analysis were used to determine odds ratio (OR) sidelong with 95% Confidence Intervals (CI). A significance level of 0.05 was used for all hypothesis tests.

## Results

### Characteristics of the participants

A total of 392 professionals from ICUs and PCUs completed the survey (67% response rate) (Fig 1). Out of these 392 professionals, 300 (77%) worked in ICUs and 92 (23%) in PCUs. The majority (69%) was women, had a median age of 32, and was non-married (55%). The sample comprised a total of 292 (74%) nurses and 100 (26%) physicians. 361 (92%) respondents were graduated in medicine/nursing and a minority was awarded with a master (6%) or a PhD (2%) degree. 107 (28%) respondents obtained post-graduate education/training in intensive/palliative care. This occurred more frequently among professionals working in PCUs (p = .031). See Table 1.

**Table 1:** Characteristics of participants. med: median. P: Percentile.

	Total (n = 392)		Setting				p
			Intensive Care		Palliative Care		
			(n = 300; 76.5%)		(n = 92; 23.5%)		
**Gender**							
Female	271	(69)	195	(65)	76	(83)	0.001*
Male	121	(31)	105	(35)	16	(17)	
**Age**, med (P25-P75)	32	(28–39)	32	(28–39)	32	(26–40)	0.848^§^
**Marital Status**							
Not married	171	(55)	139	(46)	38	(41)	0.099*
Married	177	(45)	161	(54)	54	(59)	
**Profession**							
Physician	100	(26)	82	(27)	18	(20)	0.135*
Nurse	292	(74)	218	(73)	74	(80)	
**Education level**							
Graduation degree	361	(92)	280	(94)	80	(87)	0.028*
Master	25	(6)	12	(4)	12	(13)	
Doctorate	6	(2)	6	(2)	0	(0)	
**Post-graduate education in intensive/palliative care**							
No	273	(72)	215	(75)	58	(63)	0.031*
Yes	107	(28)	73	(25)	34	(37)	

* Independent χ^2^ exact test.

^§^ Mann-Whitney Test.

Significant findings highlighted in bold.

### Burnout levels in ICUs and PCUs

A high level of burnout was identified in 27% of the respondents (19% were in high risk and 8% were experiencing burnout). 62% of the participants exhibited average and high levels of Emotional Exhaustion (EE), 60% average and high levels of Depersonalization (DEP), and 38% high levels of Personal and Professional Accomplishment (PPA). Significant differences were found between ICUs and PCUs. While 31% of the professionals working in ICUs had a high level of burnout, only 16% of the professionals working in PCUs presented that level of burnout (p = .006). Furthermore, the majority of PC practitioners (55%) exhibited a low risk of burnout; diverse findings were found among intensivists (39%), p = .022. Nevertheless, the latter group of professionals showed higher levels of PPA (42%) when compared to those working in PC (27%), p = .001. Contrariwise, the highest levels of DEP were found among professionals working in ICUs (27% of the respondents of this group reported a high level of DEP) and the lowest levels of DEP were identified among those working in PCUs (54% of the participants of this group), p = .001. No significant differences were found when comparing both settings for the sub-dimension of EE (Table 2).

**Table 2:** Results from the three dimensions of the Maslach Burnout Inventory–Human Services Survey (MBI-HSS) per setting of care (ICU vs. PCU).

			Setting				
	Total (n = 392)		PCU (n = 92; 23.5%)		ICU (n = 300; 76.5%)		p*
	n	(%)	n	(%)	n	(%)	
**Maslach Burnout Inventory–Human Services Survey (MBI-HSS)**							
**Emotional Exhaustion (EE)**							
Low (< = 14)	144	(39)	38	(43)	106	(38)	0.637
Average (15 a 24)	110	(30)	26	(29)	84	(30)	
High (> = 25)	117	(32)	25	(28)	92	(33)	
**Depersonalization (DEP)**							
Low (< = 3)	154	(40)	50	(54)	104	(36)	**0.001**
Average (4 a 9)	142	(37)	33	(36)	109	(37)	
High (> = 10)	87	(23)	9	(10)	78	(27)	
**Personal and Professional Accomplishment (PPA)**							
Low (> = 40)	104	(28)	39	(43)	65	(23)	**0.001**
Average (33 a 39)	124	(34)	27	(30)	97	(35)	
High (< = 32)	140	(38)	25	(27)	115	(42)	
**Burnout**							
Low risk	152	(43)	48	(55)	104	(39)	**0.022**
Medium risk	106	(30)	26	(30)	80	(30)	
High risk (Hr)	69	(19)	11	(13)	58	(22)	
In Burnout (InB)	28	(8)	3	(3)	25	(9)	
**High level of Burnout**							
No	258	(73)	74	(84)	184	(69)	**0.006**
Yes (Hr + InB)	97	(27)	14	(16)	83	(31)	

*Independent χ^2^ exact test.

Significant findings highlighted in bold.

In order to perform logistic regression analysis, 37 (8%) questionnaires had to be removed from our sample due to incomplete responses in the sub-dimensions of the MBI-HSS. Hence, the following results refer to a sample of 355 participants (Fig 1).

Univariate logistic regression analysis showed that the odds of exhibiting burnout were 2.384 times higher in ICUs than in PCUs. When controlling for socio-demographic, profession and workplace related variables, differences between both settings remained significant: OR = 2.525 (Table 3).

**Table 3:** Burnout in ICU versus PCU.

			Burnout						
	Total (n = 355)		No (n = 258; 73%)		Yes (n = 97; 27%)		P^¶^	OR* (95%CI)	OR** (95%CI)
**Setting**									
**PCU**	88	(25)	74	(29)	14	(14)	**0.006**	1.000	1.000
**ICU**	267	(75)	184	(71)	83	(86)		**2.384 (1.273–4.465)**	**2.525 (1.025–6.221)**

^¶^Independent χ^2^ exact test.

*Odds Ratio Univariate.

**Odds Ratio Multivariate.

CI: Confidence Interval. Significant findings highlighted in bold.

### Burnout in ICUs and PCUs: Determinant workplace conditions

Univariate regression analyses showed that, amongst others, higher burnout levels were significantly associated with the following workplace experiences: night shifts (OR = 2.232) and occurrence of conflicts (OR = 2.074) in the week prior to questionnaire completion, conflicts with other professionals in the week prior to (OR = 6.995) and day of questionnaire completion (OR = 7.176), and conflicts with patients (OR = 4.782) in the week before filling-in the survey. Burnout was also associated with experiencing a patient’s death in the day of questionnaire completion (OR = 1.866) and with ethical decision-making in the prior week. (Tables 4 and 5)

**Table 4:** Burnout related to workplace experiences in the week prior to survey completion.

			Burnout						
	Total (n = 355)		No (n = 258; 73%)		Yes (n = 97; 27%)		P	OR* (95%CI)	OR** (95%CI)
**In the week prior to survey completion:**									
**Nigh shifts**									
No	96	(28)	80	(32)	16	(18)	**0.008**^¶^	1.000	1.000
Yes	243	(72)	168	(68)	75	(82)		**2.232** **(1.223–4.075)**	**1.877** **(1.006–3.500)**
**Extra shifts**									
No	229	(69)	168	(69)	61	(69)	0.935^¶^	1.000	1.000
Yes	103	(31)	76	(31)	27	(31)		0.978 (0.577–1.659)	0.885 (0.518–1.514)
**Days-off**									
No	113	(33)	88	(35)	25	(27)	0.173^¶^	1.000	1.000
Yes	227	(67)	161	(65)	66	(73)		1.443 (0.851–2.448)	1.443 (0.846–2.461)
**Vacations/Holidays**									
No	300	(92)	216	(91)	84	(94)	0.336^¶^	1.000	1.000
Yes	26	(8)	21	(9)	5	(6)		0.612 (0.224–1.677)	0.669 (0.241–1.855)
**Patient’s death**									
No	158	(48)	122	(51)	36	(42)	0.163^¶^	1.000	1.000
Yes	169	(52)	119	(49)	50	(58)		1.424 (0.866–2.341)	1.402 (0.849–2.317)
**Number of deceased patients**									
0	158	(45)	122	(47)	36	(38)	0.257^¶^	1.000	1.000
1 a 2	141	(40)	98	(38)	43	(45)		1.487 (0.887–2.492)	1.412 (0.838–2.381)
≥3	55	(16)	38	(15)	17	(18)		1.516(0.766–2.999)	1.495(0.750–2.980)
**Conflicts**									
No	261	(79)	201	(83)	60	(70)	**0.011**^¶^	1.000	1.000
Yes	68	(21)	42	(17)	26	(30)		**2.074** **(1.175–3.659)**	**2.170** **(1.218–3.866)**
**Conflicts with:**									
**Colleagues**									
No	304	(92)	226	(93)	78	(88)	0.090^¶^	1.000	1.000
Yes	27	(8)	16	(7)	11	(12)		1.992 (0.886–4.476)	1.952 (0.859–4.432)
**Superiors**									
No	311	(93)	230	(94)	81	(91)	0.360^¶^	1.000	1.000
Yes	23	(7)	15	(6)	8	(9)		1.514 (0.619–3.705)	1.526 (0.617–3.778)
**Other Professionals**									
No	309	(93)	237	(97)	72	(81)	**<0.001**^¶^	1.000	1.000
Yes	25	(7)	8	(3)	17	(19)		**6.995** **(2.899–16.876)**	**6.701** **(2.752–16.318)**
**Patients**									
No	325	(98)	241	(99)	84	(94)	**0.034**^₸^	1.000	1.000
Yes	8	(2)	3	(1)	5	(6)		**4.782** **(1.119–20.441)**	**7.514** **(1.624–34.764)**
**Family members**									
No	307	(95)	225	(96)	82	(92)	0.262^₸^	1.000	1.000
Yes	17	(5)	10	(4)	7	(8)		1.921 (0.708–5.213)	2.779 (0.962–8.030)
**Ethical decisions**:									
**Withdrawing treatment**									
No	245	(74)	188	(77)	57	(66)	**0.041**^¶^	1.000	1.000
Yes	87	(26)	57	(23)	30	(34)		**1.736** **(1.019–2.956)**	**1.715** **(1.001–2.936)**
**Withholding treatment**									
No	220	(67)	174	(71)	46	(53)	**0.003**^¶^	1.000	1.000
Yes	110	(33)	70	(29)	40	(47)		**2.161** **(1.303–3.587)**	**2.108** **(1.263–3.518)**
**Palliative sedation**									
No	256	(78)	197	(81)	59	(69)	**0.020**^¶^	1.000	1.000
Yes	74	(22)	47	(19)	27	(31)		**1.918** **(1.101–3.343)**	1.739 (0.990–3.056)
**Communication about the diagnose/prognoses with the patient**									
No	220	(66)	155	(63)	65	(73)	0.088^¶^	1.000	1.000
Yes	115	(34)	91	(37)	24	(27)		0.629 (0.368–1.074)	0.684 (0.398–1.177)
**Communication about the diagnose/prognoses with the family**									
No	142	(42)	106	(43)	36	(41)	0.744^¶^	1.000	1.000
Yes	193	(58)	141	(57)	52	(59)		1.086 (0.663–1.780)	1.101 (0.668–1.813)

^¶^Independent χ^2^ exact test.

^§^ Mann-Whitney Test.

^₸^ Fisher exact test

*Odds Ratio Univariate.

**Odds Ratio adjusted to the setting (ICU/PCU).

CI: Confidence Interval. Significant findings highlighted in bold.

**Table 5:** Burnout related to workplace experiences in the day of survey completion.

			Burnout						
	Total (n = 355)		No (n = 258; 73%)		Yes (n = 97; 27%)		p	OR* (95%CI)	OR** (95%CI)
**In the day of survey completion:**									
**Caring for a dying patient**									
No	228	(69)	172	(71)	56	(63)	0.141^¶^	1.000	1.000
Yes	102	(31)	69	(29)	33	(37)		1.469 (0.880–2.453)	1.633 (0.965–2.764)
**Patient’s death**									
No	295	(94)	213	(93)	82	(94)	**0.011**^¶^	1.000	1.000
Yes	20	(6)	15	(7)	5	(6)		1.866 (1.305–2.459)	0.720 (0.251–2.060)
**Conflicts**									
No	325	(96)	241	(97)	84	(93)	0.116^₸^	1.000	1.000
Yes	13	(4)	7	(3)	6	(7)		2.459 (0.804–7.525)	2.629 (0.840–8.224)
**Conflicts with:**									
**Colleagues**									
No	330	(98)	241	(98)	89	(99)	0.680^₸^	1.000	1.000
Yes	7	(2)	6	(2)	1	(1)		0.451 (0.054–3.801)	0.371 (0.044–3.137)
**Superiors**									
No	331	(98)	244	(99)	87	(97)	0.196^₸^	1.000	1.000
Yes	6	(2)	3	(1)	3	(3)		2.805 (0.556–14.156)	2.663 (0.518–13.697)
**Other professionals**									
No	329	(98)	244	(99)	85	(94)	**0.007**^¶^	1.000	1.000
Yes	7	(2)	2	(1)	5	(6)		**7.176** **(1.367–37.679)**	**7.848** **(1.445–42.628)**
**Patients**									
No	331	(99)	245	(99)	86	(99)	1.000^₸^	1.000	1.000
Yes	3	(1)	2	(1)	1	(1)		1.424 (0.128–15.907)	1.188 (0.106–13.304)
**Family members**									
No	331	(99)	244	(100)	87	(99)	0.459^₸^	1.000	1.000
Yes	2	(1)	1	(0)	1	(1)		2.805 (0.174–45.325)	3.541 (0.205–61.306)
**Ethical decisions:**									
**Withdrawing treatment**									
No	309	(92)	228	(93)	81	(90)	0.353^¶^	1.000	1.000
Yes	26	(8)	17	(7)	9	(10)		1.490 (0.639–3.475)	1.567 (0.662–3.705)
**Withholding treatment**									
No	298	(89)	214	(88)	84	(92)	0.232^¶^	1.000	1.000
Yes	37	(11)	30	(12)	7	(8)		0.594 (0.251–1.406)	0.606 (0.254–1.445)
**Palliative sedation**									
No	308	(92)	224	(91)	84	(93)	0.570^¶^	1.000	1.000
Yes	27	(8)	21	(9)	6	(7)		0.762 (0.297–1.953)	0.804 (0.310–2.083)
**Communication about the diagnose/prognoses with the patient**									
No	285	(85)	210	(85)	75	(82)	0.506^¶^	1.000	1.000
Yes	52	(15)	36	(15)	16	(18)		1.244 (0.653–2.372)	1.387 (0.717–2.683)
**Communication about the diagnose/prognoses with the family**									
No	244	(72)	179	(73)	65	(71)	0.808^¶^	1.000	1.000
Yes	93	(28)	67	(27)	26	(29)		1.069 (0.626–1.824)	1.118 (0.650–1.922)

^¶^Independent χ^2^ exact test.

^§^ Mann-Whitney Test.

^₸^ Fisher exact test

*Odds Ratio Univariate.

**Odds Ratio adjusted to the setting (ICU/PCU).

CI: Confidence Interval. Significant findings highlighted in bold.

When adjusting for the setting (i.e., ICUs/PCUs), night shifts and experiencing conflicts in the week prior to survey completion were associated with higher burnout levels (OR = 1.877 and 2.170, respectively). Certain experiences in the week before participating in the survey also remained significantly associated to burnout, namely: conflicts with other professionals (OR = 6.701), conflicts with patients (OR = 7.514), withholding treatment (OR = 2.108), and withdrawing treatment (OR = 1.715). Conflicts with other professionals in the day of questionnaire completion remained significant and positively associated to burnout (OR = 7.848). (Tables 4 and 5)

When controlling for socio-demographic and educational characteristics of the participant professionals and for the setting (ICUs vs. PCUs) in the multivariate logistic regression analyses, the only variables that remained significantly associated to burnout were 'conflicts' and having post-graduate education in IC/PC. Experiencing conflicts increased the odds of exhibiting burnout 3.124 times and having post-graduate education diminished its likelihood (OR = 0.395). See Table 6.

**Table 6:** Burnout in ICU and PCU related to socio-demographic, professional and workplace experiences in the week prior to survey completion. med-median. P-Percentile.

			Burnout							
	Total (n = 355)		No (n = 258; 73%)		Yes (n = 97; 27%)		p	OR* (95%CI)	OR** (95%CI)	OR*** IC 95% N = 288 (81%)
**Setting**										
PCU	88	(25)	74	(29)	14	(14)	**0.006**^¶^	1.000	-	1.000
ICU	267	(75)	184	(71)	83	(86)		**2.384** **(1.273–4.465)**	-	**2.525** **(1.025–6.221)**
**Gender**										
Female	245	(69)	178	(69)	67	(69)	0.988^¶^	1.000	1.000	1.000
Male	110	(31)	80	(31)	30	(31)		0.996 (0.601–1.651)	0.886 (0.530–1.483)	0.719 (0.360–1.440)
**Age** med (P25-P75)	32	(27–38)	33	(28–40)	30	(27–35)	**0.004**^§^	**0.955** **(0.925–0.986)**	**0.955** **(0.924–0.986)**	0.915 (0.764–1.097)
**Marital Status**										
Single	158	(45)	106	(41)	52	(54)	0.106^¶^	1.000	1.000	1.000
Married	156	(44)	120	(47)	36	(37)		0.612 (0.371–1.007)	0.610 (0.368–1.009)	1.136 (0.502–2.573)
Divorced/Widow/Other	41	(12)	32	(12)	9	(9)		0.573 (0.255–1.289)	0.631 (0.277–1.435)	0.872 (0.270–2.817)
**Profession**										
Physician	91	(26)	73	(28)	18	(19)	0.061^¶^	1.000	1.000	1.000
Nurse	264	(74)	185	(72)	79	(81)		1.732 (0.970–3.091)	**1.849** **(1.029**–**3.321)**	1.673 (0.494–5.666)
**Post-graduate education in intensive/palliative care**										
No	248	(72)	177	(71)	71	(76)	0.285^¶^	1.000	1.000	1.000
Yes	96	(28)	74	(29)	22	(24)		0.741 (0.428–1.284)	0.808 (0.462–1.412)	**0.395** **(0.178–0.878)**
**Shift work**										
No	68	(19)	56	(22)	12	(12)	**0.045**^¶^	1.000	1.000	1.000
Yes	286	(81)	201	(78)	85	(88)		**1.973** **(1.007–3.868)**	1.660 (0.833–3.311)	0.947 (0.351–2.555)
**Nr. of working hours per week**										
35 hours	117	(33)	89	(35)	28	(29)	**0.029**^¶^	1.000	1.000	1.000
40 hours	125	(35)	79	(31)	46	(47)		**1.851** **(1.058–3.237)**	**1.896** **(1.079–3.330)**	1.797 (0.638–5.066)
42 hours	55	(16)	43	(17)	12	(12)		0.887 (0.412–1.912)	0.949 (0.437–2.060)	2.124 (0.653–6.912)
Other	57	(16)	46	(18)	11	(11)		0.760 (0.347–1.663)	1.036 (0.453–2.367)	1.527 (0.483–4.828)
**In the week prior to survey completion:**										
**Night shifts**										
No	96	(28)	80	(32)	16	(18)	**0.008**^¶^	1.000	1.000	1.000
Yes	243	(72)	168	(68)	75	(82)		**2.232** **(1.223–4.075)**	**1.877** **(1.006–3.500)**	0.902 (0.421–1.929)
**Patient’s death**										
No	158	(48)	122	(51)	36	(42)	0.163^¶^	1.000	1.000	1.000
Yes	169	(52)	119	(49)	50	(58)		1.424 (0.866–2.341)	1.402 (0.849–2.317)	0.950 (0.462–1.953)
**Conflicts**										
No	261	(79)	201	(83)	60	(70)	**0.011**^¶^	1.000	1.000	1.000
Yes	68	(21)	42	(17)	26	(30)		**2.074** **(1.175–3.659)**	**2.170** **(1.218–3.866)**	**3.124** **(1.475–6.619)**
**Ethical decisions**										
**Withdrawing treatment**										
No	245	(74)	188	(77)	57	(66)	**0.041**^¶^	1.000	1.000	1.000
Yes	87	(26)	57	(23)	30	(34)		**1.736** **(1.019–2.956)**	**1.715** **(1.001–2.936)**	1.360 (0.536–3.448)
**Withholding treatment**										
No	220	(67)	174	(71)	46	(53)	**0.003**^¶^	1.000	1.000	1.000
Yes	110	(33)	70	(29)	40	(47)		**2.161** **(1.303–3.587)**	**2.108** **(1.263–3.518)**	1.865 (0.840–4.142)
**Palliative sedation**										
No	256	(78)	197	(81)	59	(69)	**0.020**^¶^	1.000	1.000	1.000
Yes	74	(22)	47	(19)	27	(31)		**1.918** **(1.101–3.343)**	1.739 (0.990–3.056)	1.221 (0.494–3.022)

^¶^Independent χ^2^ exact test.

^§^ Mann-Whitney Test.

*Odds Ratio Univariate.

**Odds Ratio adjusted to the setting (ICU/PCU).

***Odds Ratio adjusted to all variables present in the table.

CI: Confidence Interval. Significant findings highlighted in bold.

## Discussion

Our results show that a substantial proportion of professionals working in ICUs and PCUs are at high risk of developing burnout and that the likelihood of developing this syndrome is significantly higher in intensive care units (ICUs) when compared to palliative care units (PCUs). Developing burnout is associated with several workplace conditions, such as experiencing conflicts (e.g., with patients and/or families, intra and/or inter-teams). Post-graduate education plays a relevant role in diminishing the risk of burnout.

### Burnout in ICUs and PCUs: Does it make a difference?

A significant percentage of physicians and nurses working in ICUs and PCUs either were experiencing burnout or were at high risk of developing burnout. This is of foremost relevance as it means that these professionals were exhausted, established cold, cynical and distant relationships and experienced a reduced sense of personal and professional accomplishment [2,3], thus affecting the quality of interactions, inter-professional relations and care provision. Indeed, our findings pinpointed that a large percentage of professionals had high levels of depersonalization. Patients suffering from life-threatening diseases, at the very end-of-life, are in a condition of vulnerability. This vulnerability may be increased if these patients are being cared for by professionals who are themselves affected in their health status, as high levels of burnout are related to health problems such as depressive symptoms [31–35].

As described elsewhere [1], the ethical dimension and framework to understand burnout is related to its consequences, especially at two levels: (i) the increase in the patient’s vulnerability owing to the consequences that burnout may have for patients and their relatives; and (ii) the increase in professionals’ vulnerability due to their condition of suffering from a work-related syndrome affecting their own health. A third level of the ethical framework to understand burnout refers to the principle of responsibility in terms of burnout prevention at individual (to take care of oneself), team (to take care for oneself and for the others) and organizational (e.g., organizational ethics, creating empowering workplace conditions) levels [1].

In what refers to the sub-dimensions of burnout, our findings are a source of major concern. First, the large proportion of professionals experiencing emotional exhaustion constitutes a risk to patient safety and for the quality of care. Burnout was identified as a predictor of reporting major medical errors by physicians [36–43], affecting patients’ safety and wellbeing. This, however, is not consensual. A recent study showed that medical errors were associated to depressive symptoms among ICU staff but not to burnout [44]. In a six-country cross-national study, it was found that burnout in nurses was associated with reduced quality of care, lower patient satisfaction and increased errors [45]. Second, the consequences of depersonalization may undermine the relationship, communication and inter-connection established among professionals, patients and families. Evidence shows that high levels of depersonalization were associated with self-reported sub-optimal care provision [46] and with a sense of not dedicating enough time to patients [39]. Third, the consequences of a lack of personal and professional accomplishment may affect the whole care system at micro, meso and macro levels due to absenteeism, turnover, and intention to leave [6,12,13,31,47]. In fact, excessive turnover rates increase healthcare costs, decrease productivity, lower staff morale, and reduce the overall quality of care, because experienced professionals who leave their workplaces must be replaced [13,47–50]. Considering the major financial crisis and its effects in the Portuguese healthcare system, this is particularly important. For instance, in Canada, a recent study showed that the total cost of burnout among Canadian physicians was estimated to be more than $200 million [51]. In the United States, replacement costs have been estimated to be at least $250,000 for each primary care physician, an amount certainly higher for other medical specialties, namely intensive and palliative care [52]. It is imperative not only to consider the effect of constrained resources in the shortage of the healthcare workforce, but also to implement strategies aiming at improving organizational structures and processes that can reduce the micro-meso-macro consequences of burnout and increase the satisfaction and wellbeing of healthcare professionals [4,48].

Our findings also showed that working in ICUs more than doubled the likelihood of having burnout when compared to working in PCUs. These findings are similar to those found in other studies, suggesting that burnout in PCUs is comparable or lower than in other healthcare settings [1,9,53,54]. Possible explanations were provided by several authors who emphasize the meaning given by PC professionals and teams to caring for patients facing life-threatening diseases and promoting their wellbeing and quality of life. This contributes to the development of personal awareness and gives a sense of gratification [1,18,19,55–59]. Furthermore, by being one of the dimensions of healthcare professional empowerment, meaning is paramount as it gives a sense of purpose or personal connection about the care provided in the workplace [6,59].

In addition, it seems that PC teams embrace active strategies and rituals when facing a patient’s death, helping them to cope with those losses [1,55–57,60]. Several authors have emphasized that professionals working in palliative care usually make additional recommendations on how to prevent and deal with burnout. These professionals are encouraged to focus on spirituality and human nature, methods for self-care including taking regular breaks from work and focusing on the positive aspects of life, so that one is not overwhelmed by adversity and suffering [61–64].

Contrarily, time pressure in ICUs may damper communication within the team and between professionals and patients. This may increase stress, tensions and conflicts that can boost the risk of developing burnout [9,13,14,47,65–69].

### Burnout in ICUs and PCUs: What can make a difference?

Several workplace factors (e.g., night shifts, patient’s death, conflicts, and ethical decisions) were associated with burnout. These findings are aligned with other studies [4,12,16,31,47,66] showing the impact of socio-structural workplace conditions on professionals’ wellbeing. However, when controlling for socio-demographic, setting, and workplace variables, the only variables that remained significantly associated with burnout were conflicts and having post-graduate education in intensive/palliative care. While experiencing conflicts strongly increased the likelihood of burnout, having post-graduate education diminished its chances.

This is not surprising since the occurrence of conflicts at work was identified as a major burnout risk factor in the literature [4,14,16,31,47,66]. We cannot ensure, however, that there is a cause-effect relationship in which conflicts cause burnout. It can also occur that professionals with burnout more often experience conflicts. Conflicts result mainly from disagreements about the goals of treatment and care pathways, the philosophy of care, ethical decision-making, end-of-life care, and inter-professional relations [4,47,65,66,70–74]. Hence, it is paramount to ensure and promote inter-professional and team-based strategies (e.g., team meetings, quality improvement initiatives) aiming at improving communication, organization and sharing visions. Well-led meetings may promote a sense that all team members “have a voice” and contribute equally to the chain-of-events affecting patient and family care outcomes, professionals’ wellbeing, and conflicts [73–76]. This is a current practice, part of daily dynamics in PCUs and a protective factor against burnout [5,18,76,77]. Inter-professional team-based working strategies from PCUs may therefore be translated into ICUs, helping to overcome some burnout-associated factors (e.g., conflicts and end-of-life decision-making). Involving the interdisciplinary team may indeed leverage aspects of interaction among professionals that may ameliorate burnout [5,78,79].

Finally, our findings show that post-graduate education in intensive/palliative care plays a relevant role in protecting professionals from burnout and happened more frequently among PC professionals. This is in line with other studies showing that post-graduate education or other specific education programs were inversely associated to burnout [1,5,16,18] and highlights the need to further implement education programs in intensive/palliative care as part of the integration process of professionals in ICUs and PCUs. Professionals with higher levels of education are able to develop their awareness about the purposes of care provision in their field of practice. Through education, death may not be seen as frustration or failure [5,18,80] and communication and end-of-life decision-making may be improved; for instance, by providing education about PC to professionals working in ICUs [81,82]. By creating awareness, education about palliative care may also increase healthcare professionals’ perceptions of self-efficacy and empowerment. This has been identified as a determinant factor of self-perceived effectiveness among healthcare professionals [6]. Furthermore, a recent systematic review has shown that interventions aimed at improving the wellbeing of staff working in palliative care settings comprised a mixture of activities, including education [83]. The interdisciplinary approaches used in most of the post-graduate programs in IC/PC may foster the development of inter-professional communication competencies, contributing to conflict management. It can also be that post-graduate education programs in palliative care provide healthcare professionals with “managing people and change” competencies, which enhance perceptions of empowerment and effectiveness in the workplace [5,6].

### Strengths, limitations and further research

A major strength of our study is its originality and relevance to a wide audience when comparing burnout among healthcare professionals providing end-of-life care in two specific settings: ICUs and PCUs. To our best knowledge, this is the first study making such a comparison. Hence, our findings contribute to the existing literature about burnout in end of life care. Furthermore, it provides relevant information that may foster an understanding and a reflection on the potential and need of integrating palliative care approaches and practices into intensive care. This calls for further research on the best integration models and their effectiveness for patient and family-related outcomes, for professionals and teams’ well-being and for the resilience and sustainability of healthcare services and systems.

Nevertheless, a few limitations need to be considered. First, the study used a cross-sectional design and a nonrandomized sampling procedure. The geographic location of the participant units varied (while palliative care units were located nationwide, intensive care units were situated only in the North of the country). This makes the generalization of findings limited. Second, lower burnout levels in palliative care need to be seen with some caution. It is relevant to note, however, that we obtained a considerably high response rate (approximately 70% of the potential participants completed the survey). Moreover, previous research conducted with the same palliative care units [5,18,57] support our findings. Third, the subjectivity inherent to the concept of conflict may be considered. Since we aggregated all types of conflicts (intra-team, inter-teams, with a superior, with patients, with family members) for the multivariate analysis, more research is needed to complement the understanding of this variable significantly associated to burnout. Finally, while we used internationally and Portuguese validated definition criteria and cut-off scores to assess burnout [3,21–26], it is worth mention that accurate specific cut-off values for US critical care professionals have not been determined yet [9,13]; the same occurs for palliative care professionals. Nevertheless, international evidence indicates that the MBI-HSS seems to be quite usable across diverse national and cultural work settings. In fact, it is striking that results from different countries both in the United States and in Europe are so similar [27–30].

The results of this study highlight the need for a better and regular assessment of professional burnout and its associated organizational factors and workplace experiences in palliative and intensive care. Further research is needed to assess the organizational culture and the factors needed to sustain healthy work environments. In our opinion, these studies should provide an in-depth and meaningful understanding on what and how personal characteristics of the professionals, specific conflicts, organizational and socio-structural factors of the workplace and situations associated to end-of-life care in these settings may increase or prevent burnout.

## Conclusions

In this study, a high level of burnout was identified in 27% of the professionals. Working in intensive care units more than doubled the likelihood of exhibiting burnout when compared to working in palliative care units. Experiencing conflicts was the most significant determinant of burnout and post-graduate education in intensive/palliative care protected professionals from developing this syndrome. This highlights the need for promoting empowering workplace conditions, such as education programs, leadership, team empowerment and conflict management. The use of a “toolbox” for early assessment of burnout and the promotion of empowering workplace conditions (e.g., interdisciplinary teamwork, teamwork practices and dynamics, and conflict management) can be determinant for burnout prevention and minimization. These and other strategies aimed at improving organizational structures and processes can reduce the micro-meso-macro consequences of burnout and increase the satisfaction and wellbeing of healthcare professionals. Some of these team-based strategies are a current practice in palliative care and may therefore be translated into intensive care, helping to overcome some burnout-associated factors (e.g., conflicts and ethical decisions at the end of life). In sum, findings suggest the need for implementing quality improvement and organizational redesign strategies aimed at integrating the philosophy, principles and practices of palliative care in intensive care units.

## Abbreviations

CI: Confidence Intervals
DEP: Depersonalization
EE: Emotional Exhaustion
IC: Intensive Care
ICUs: Intensive Care Units
MBI-HSS: Maslach Burnout Inventory—Human Services Survey
OR: Odds Ratio
PC: Palliative Care
PCUs: Palliative Care Units
PPA: Personal and Professional Accomplishment
